# Dimethyl Sulfoxide Enhances HLA Peptide Identification

**DOI:** 10.3390/proteomes14010013

**Published:** 2026-03-13

**Authors:** Terry C. C. Lim Kam Sian, Yue Ding, Scott A. Blundell, Ralf B. Schittenhelm, Pouya Faridi

**Affiliations:** 1 Centre for Cancer Research, Hudson Institute of Medical Research, Clayton, VIC 3168, Australia; 2 Department of Medicine, School of Clinical Sciences, Monash University, Clayton, VIC 3168, Australia; 3 Monash Proteomics and Metabolomics Platform, Clinical Proteomics Node, School of Clinical Sciences, Monash University, Clayton, VIC 3168, Australia; 4 Monash Proteomics and Metabolomics Platform, Monash Biomedicine Discovery Institute, Monash University, Clayton, VIC 3168, Australia

**Keywords:** DMSO, HLA peptides, immunopeptidomics, electrospray ionisation

## Abstract

**Background:** Mass spectrometry (MS)-based immunopeptidomics has emerged as the gold standard for profiling HLA-bound peptides, yet detection remains challenging due to their non-tryptic nature, variable lengths, and lack of basic residues, which limit ionisation and fragmentation efficiency. **Methods:** To address these limitations, we investigated the impact of incorporating 5% dimethyl sulfoxide (DMSO) into LC-MS/MS mobile-phase buffers on immunopeptidomic workflows. Using B-lymphoblastoid cell lines expressing HLA class I and II alleles and elastase-digested HeLa lysates as a surrogate for non-tryptic peptides, we assessed peptide identification, ionisation efficiency, charge state distribution, and fragmentation quality. **Results:** DMSO significantly increased peptide identifications across all sample types, with gains of ~1.33 folds for HLA class I, ~1.55 folds for HLA class II, and ~1.24 folds for elastase digests. Improvements were systematic and reproducible, driven by enhanced electrospray ionisation, higher charge states, and superior MS2 spectral quality, evidenced by ~2-fold increase in b- and y-ion intensities. Importantly, DMSO did not introduce major sequence bias, preserving motif integrity and predicted binding characteristics. **Conclusions:** Overall, these findings establish DMSO as a robust additive for improving sensitivity and reliability in immunopeptidomics, particularly for low-input or clinically derived samples.

## 1. Introduction

Immunopeptidomics is a technology for the comprehensive identification and characterisation of peptides presented on the cell surface by major histocompatibility complex (MHC) molecules, specifically human leukocyte antigens (HLAs). These peptides are central to antigen presentation and play a pivotal role in modulating adaptive immune responses, influencing processes such as immune surveillance, tumour recognition, and pathogen clearance [1,2]. Consequently, the immunopeptidome provides a crucial molecular framework for the development of vaccines, immunotherapies, and diagnostic biomarkers [3].

Mass spectrometry (MS)-based immunopeptidomics has emerged as the gold standard for profiling HLA-bound peptides due to its sensitivity and ability to detect a wide range of peptide species [4]. However, the detection of HLA peptides remains technically challenging. Unlike tryptic peptides, which terminate in basic amino acids (arginines and lysines) that promote efficient ionisation during electrospray ionisation (ESI), HLA peptides often lack these C-terminal residues [5]. This results in suboptimal charge state distributions, reduced ionisation efficiency, and lower selection for fragmentation, ultimately limiting peptide identification [6].

These challenges are further compounded by the intrinsic diversity of the immunopeptidome. HLA-associated peptides display substantial heterogeneity in sequence length, amino acid composition, and binding motifs, reflecting the extensive polymorphism and distinct peptide-binding specificities of HLA molecules. HLA class I peptides are typically short (8–12 amino acids) and constrained by defined anchor residues at fixed positions within a closed binding groove, whereas HLA class II peptides are generally longer (13–20 amino acids) and bind within an open-ended groove, allowing variable peptide lengths and multiple binding motifs. This structural complexity translates into a broad physicochemical landscape, encompassing differences in charge distribution, hydrophobicity, and gas-phase behaviour during MS analysis, and further complicates chromatographic separation, ionisation, and fragmentation.

Supercharging agents have been explored in the broader field of mass spectrometry to enhance ionisation efficiency and improve peptide detection. Among these, dimethyl sulfoxide (DMSO) has emerged as a promising additive. DMSO has been shown to increase the charge state of proteins and peptides during ESI, likely due to its high surface tension and gas-phase basicity [7]. It can induce protein unfolding in ESI droplets, thereby exposing more protonation sites and enhancing charge states [8]. These effects have been demonstrated in various proteomic contexts, including the analysis of tryptic peptides, chymotryptic peptides, monoclonal antibodies, and protein complexes [9,10].

Despite its widespread use in general proteomics, the application of DMSO in immunopeptidomics remains underexplored. Given the non-tryptic nature, structural diversity, and often low abundance of HLA-associated peptides, strategies that enhance ionisation and stabilise higher charge states may be particularly beneficial for immunopeptidomic analyses. In this context, DMSO represents a simple and potentially effective approach to improve sensitivity and depth of coverage across both HLA class I and class II peptide repertoires.

Here, we investigated the impact of incorporating DMSO into LC-MS/MS mobile-phase buffers to enhance the identification of HLA-bound peptides. We assessed its effects on peptide ionisation, charge state distribution, fragmentation efficiency, and overall peptide identification yield across HLA class I and II molecules. Additionally, elastase-digested HeLa lysates were used as a surrogate model to mimic the complexity of non-tryptic HLA-like peptides. Our findings demonstrate that DMSO significantly improves the detection and identification of HLA peptides across both class I and class II molecules, with a more pronounced effect observed for class I. This enhancement is achieved by improving electrospray ionisation and spectral quality, without introducing sequence bias or compromising data integrity. These results highlight the potential of DMSO as a valuable additive to improve the sensitivity and robustness of immunopeptidomic analyses, especially in the case of HLA class I.

## 2. Materials and Methods

### 2.1. Cell Culture

B-lymphoblastoid cell lines (B-LCLs) expressing HLA class I alleles (HLA-A*01:01, HLA-B*08:01, and HLA-C*07:01) as well as class II alleles (HLA-DRB1*01:01, HLA-DPA1*01:03, and HLA-DQA1*01:02) were cultured in T25 flasks at 37 °C in a humidified atmosphere containing 5% CO_2_. Cells were maintained in complete tumour stem medium (TSM), which was supplemented with 10 ng/mL platelet-derived growth factor-AA (PDGF-AA) and 10 ng/mL PDGF-BB to support proliferation [11]. TSM was prepared by combining Neurobasal-A (GIBCO, Grand Island, NY, USA, #10888022) and DMEM/F-12 (GIBCO, #10565018) at a 1:1 ratio and supplementing with the following components: 10 mM HEPES (GIBCO, #15630080), 1 mM sodium pyruvate (GIBCO, #11360070), 1× MEM non-essential amino acids (GIBCO, #11140050), 1× GlutaMAX (GIBCO, #35050061), 1% penicillin–streptomycin (GIBCO, #15070063), 1× B27 supplement (minus vitamin A) (Thermo Fisher Scientific, Waltham, MA, USA, #12587010), 20 ng/mL epidermal growth factor (EGF) (Jomar Life Research, Scoresby, VIC, Australia, #100-26-100μg), 20 ng/mL fibroblast growth factor-basic (FGF-basic) (Jomar Life Research, #100-146-100μg), 10 ng/mL PDGF-AA (Stemcell Technologies, Vancouver, BC, Canada, #78095.2), 10 ng/mL PDGF-BB (Stemcell Technologies, #78097.1), and 2 µg/mL heparin (Stemcell Technologies, #7980). The cells were passaged upon reaching 90% confluency. The monolayer was washed with phosphate-buffered saline (PBS) (GIBCO, #14190144), treated with 1 mL of Accutase (Sigma, St. Louis, MO, USA, #A6964), and incubated at 37 °C for 3 min. The enzymatic reaction was quenched with 3 mL of PBS, followed by centrifugation at 250× *g*. 5 × 10^7^ cells were resuspended in 1 mL of pre-warmed TSM and reseeded into 5 mL of fresh medium. For downstream applications, cell pellets were harvested by centrifugation at 2500× *g* for 15 min at 4 °C, snap-frozen in liquid nitrogen, and stored at −80 °C.

### 2.2. Preparation of Elastase-Digested Peptides

HeLa cells were lysed in 1% sodium deoxycholate (SDC) (Sigma, #D6750) in 100 mM Tris-HCl (pH of 8.1), boiled at 95 °C for 5 min, and sonicated. Protein concentration was quantified, and 5 mg of total protein was subjected to reduction with 10 mM TCEP (Thermo Fisher Scientific, #77720) and alkylation with 40 mM chloroacetamide (Sigma, #C0267). After a second boiling step, the pH was adjusted to ~8.0, and proteins were digested overnight at 37 °C using elastase (1:20 enzyme-to-substrate ratio) (Promega, Madison, WI, USA, #V1891). Peptides were desalted using C18 solid-phase extraction (Waters, Milford, MA, USA, #WAT036810) and eluted with 50% acetonitrile (ACN)/0.1% trifluoroacetic acid (TFA) (Thermo Fisher Scientific, #FSBA116).

### 2.3. Isolation of HLA-Peptide Complexes

HLA-peptide complexes were isolated using SAPrIM method in a semi-automated fashion using the Kingfisher Duo (Thermo Scientific, Waltham, MA, USA), as previously described [12]. Briefly, the B-LCL pellets were lysed in 500 µL of lysis buffer composed of 1% CHAPS Detergent (3-((3-cholamidopropyl) dimethylammonio)-1-propanesulfonate) (Thermo Scientific, #28299), 50 mM Tris-HCl (pH 8.0), 150 mM NaCl, 10 mM 2-chloroacetamide (Sigma, #C0267), and 1× Halt™ Protease and Phosphatase Inhibitor Cocktail (Thermo Scientific, #78430). Lysates were gently mixed and incubated on a rotating platform at 4 °C for 1 h to ensure efficient solubilisation of membrane-bound HLA complexes. Immunoprecipitation was performed using hyper-porous magnetic Protein A beads (MagReSyn^®^, ReSyn Biosciences, Johannesburg, South Africa, #MR-PRA) conjugated with the pan-HLA class I monoclonal antibody W6/32 (Leinco Technologies, Fenton, MO, USA, #H263). Following the affinity purification, peptide ligands were eluted using 0.1% trifluoracetic acid (TFA) (Thermo Fisher Scientific, #FSBA116) and desalted using C18 solid-phase extraction columns (BioPureSPN, Hamilton Company, Reno, NV, USA, #HMM S18V) to remove detergents, salts, and other interfering substances prior to mass spectrometry analysis. The peptides were then resuspended in MS loading buffer (2% ACN/0.1 FA) to generate 6 technical replicates for mass spectrometry analysis (3 replicates for control and 3 replicates for DMSO).

### 2.4. LC-MS/MS of Peptide Samples

Peptide samples were analysed using a data-dependent acquisition (DDA) strategy on an Orbitrap HF mass spectrometer (Thermo Fisher Scientific) coupled to an UltiMate™ 3000 RSLCnano UHPLC system as previously described [13]. Prior to analysis, peptides were reconstituted in 2% acetonitrile (ACN) (Thermo Fisher Scientific, #FSBA955) with 0.1% trifluoroacetic acid (TFA) (Thermo Fisher Scientific, #FSBA116) and loaded onto a trap-and-elute configuration consisting of a PepMap™ 100 C18 trap column (50 mm × 300 µm, 5 µm particle size, 100 Å pore size) (Thermo Fisher Scientific, #164750) and a PepMap™ 100 C18 analytical column (50 cm × 75 µm, 2 µm, 100 Å) (Thermo Fisher Scientific, #164942). Peptide separation was achieved using a binary gradient consisting of a 90 min linear step from 6% to 36% buffer B (80% ACN, 0.1% formic acid (Thermo Fisher Scientific, #FSBA117)), followed by a ramp to 99% buffer B at 103 min, which was held for 5 min. When applicable, 5% dimethyl sulfoxide (DMSO) (Thermo Fisher Scientific, #85190) was added to both mobile-phase buffers A (0.1% formic acid) and B. The flow rate was maintained at 250 nL/min. Full MS (MS1) scans were acquired over an *m*/*z* range of 350–1700 at a resolution of 120,000, with an RF lens setting of 40%. The automatic gain control (AGC) target was set to 250%, with a maximum injection time of 50 ms. Precursor ions with charge states from +1 to +5 were selected for fragmentation, and dynamic exclusion was applied for 10 s within a 10 ppm mass tolerance. For MS/MS (MS2) scans, precursor ions were isolated using a 1.1 *m*/*z* window and fragmented via higher-energy collisional dissociation (HCD) at a normalised collision energy of 30%. Fragment ions were detected at a resolution of 15,000, with the scan range mode set to “define first mass” at 110 *m*/*z*. The AGC target was set to 200%, with a maximum injection time of 100 ms.

### 2.5. Data Analysis and Processing

The mass spectrometry raw files were analysed using PEAKS 12 (Bioinformatics Solutions Inc., Waterloo, ON, Canada) using the following parameters [14]: a precursor mass tolerance of 10 ppm and a fragment ion mass tolerance of 0.02 Da. An initial de novo sequencing step was performed to generate candidate peptide sequences, followed by a database search against the UniProt human proteome (v2021). The search parameters for HLA peptides included oxidation of methionine (M), N-terminal acetylation of lysine (K), and carbamidomethylation of cysteine (C) as variable modifications, while for elastase-digested samples, carbamidomethylation of cysteine (C) was applied as a fixed modification. A false discovery rate (FDR) threshold of 1% was applied at the PSM level to ensure high-confidence identifications, and all peptides passing this threshold were exported for downstream analysis (Supplementary Table S1).

### 2.6. HLA Binding Prediction

Binding affinity predictions for HLA class I (8–12mer) and class II (13–20mer) peptides were performed using the NetMHCpan 4.1 and NetMHCIIpan 4.1 supervised algorithms, respectively [15,16,17]. Peptides were classified as binders based on default rank thresholds: peptides with a rank ≤2% were considered HLA class I binders, and those with a rank ≤5% were considered HLA class II binders. The complete set of predicted binding affinities is provided in Supplementary Table S2. Unsupervised peptide clustering was performed using GibbsCluster 2.0 [18].

## 3. Results

### 3.1. DMSO Enhances the Number of HLA Class I and Class II Peptides

**Figure 1:**
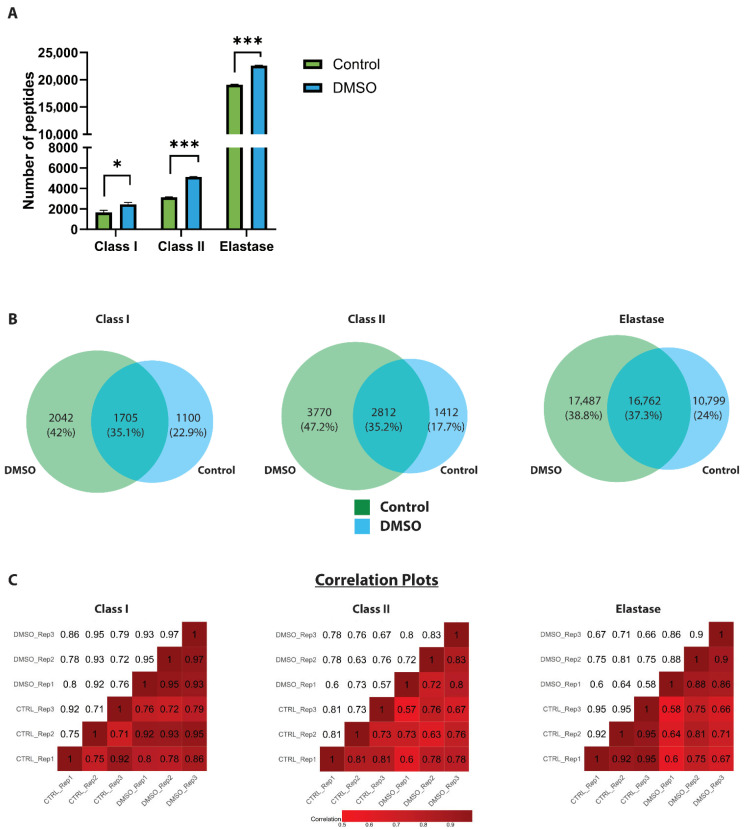
The effect of DMSO on the identification of HLA-I and HLA-II bound peptides. (**A**) Bar charts summarising the number of unique peptides in HLA Class I, HLA Class II and elastase-digested samples between control (green) and DMSO (blue) conditions. (**B**) Venn diagrams illustrating the overlap in the peptide repertoire between control (green) and DMSO (blue) conditions. (**C**) Pearson correlation plots of peptide abundance, demonstrating strong reproducibility across replicates for all sample types and consistent detection under both conditions, demonstrating strong reproducibility across replicates for all sample types, indicating consistent peptide detection in both control and DMSO-treated conditions for HLA Class I, HLA Class II, and elastase-digested samples. Statistical analysis was performed with the Student t-test. ns: non-significant, *: <0.05 and ***: <0.0001. Data represent the mean ± standard error of the mean (SEM) from three technical replicates (N = 3).

To assess the effect of DMSO on HLA peptide identification, we analysed identical immunopeptidomic samples by high-resolution LC-MS/MS with and without the addition of 5% DMSO to both mobile-phase buffer systems. Incorporating DMSO resulted in a distinct increase in the number of unique peptides, with HLA class I and class II showing gains of ~1.33 and ~1.55 folds, respectively (Figure 1A,B). A comparable, increase of ~1.24 folds was observed in an elastase-digested HeLa lysate used as a surrogate for HLA-like, non-tryptic peptides. Importantly, these increases were highly consistent across replicates, as evidenced by small standard deviations in peptide counts and strong inter-replicate correlations in peptide abundance profiles. (Figure 1A,C). Analysis of the replicate-level overlap further confirmed expected levels of reproducibility: for HLA class I, the mean peptide overlap across the three replicates was 21% under control conditions and 30% with DMSO, while HLA class II replicates showed ~40% overlap in both conditions—values consistent with typical DDA-based immunopeptidomics experiments (Supplementary Figure S1).

Peptide rank-abundance analysis revealed overall increased signal intensities in the presence of DMSO (Supplementary Figure S2), indicating that the higher number of peptide identifications is primarily driven by improved sensitivity. On the other hand, comparative analyses revealed that the shared peptide repertoire under control and DMSO conditions was relatively modest (35–38%), with a notable fraction of peptides (17–24%) exclusively identified under control conditions (Figure 1B). Nevertheless, a substantially larger fraction (40–48%) was uniquely detected in the presence of DMSO (Figure 1B), indicating that DMSO enhanced overall peptide yield by enabling the detection of a distinct subset of peptides that remained undetected under control conditions. It should be noted that when viewed from the control perspective, 51–67% of control-derived HLA class I and class II peptides were also detected under DMSO conditions, which is higher than what the Venn diagram alone might suggest—highlighting that a larger share of control peptides was retained in the DMSO dataset (Figure 1B). Collectively, these findings establish 5% DMSO as an effective additive for immunopeptidomic LC-MS/MS workflows, capable of significantly expanding immunopeptidome coverage for both HLA class I and II peptides while maintaining reproducibility and robustness.

### 3.2. DMSO Favours Shorter Peptides

**Figure 2:**
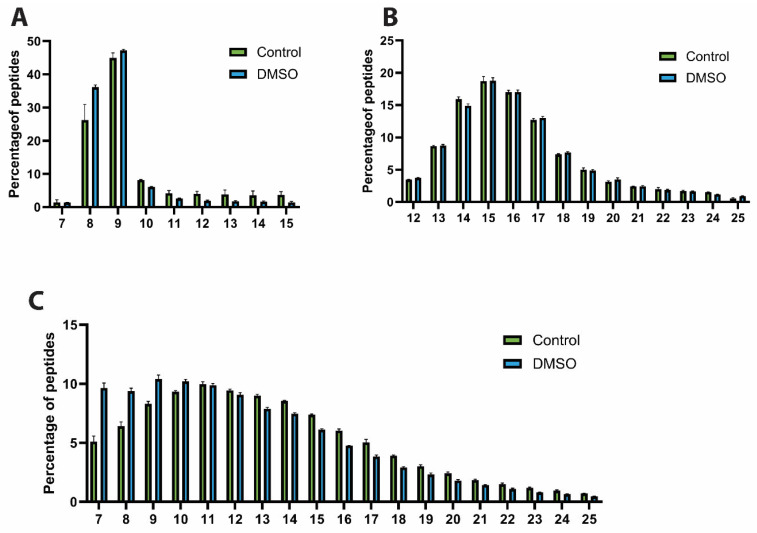
Peptide length distribution. Bar charts showing the distribution of peptide lengths for (**A**) HLA Class I, (**B**) HLA Class II, and (**C**) elastase-digested peptides under control (green) and DMSO-treated (blue) conditions. The plots highlight the relative enrichment of shorter peptides in the presence of DMSO, particularly within HLA Class I and elastase samples. Data represent the mean ± standard error of the mean (SEM) from three technical replicates (N = 3).

To assess the characteristics of the identified HLA peptide repertoire and to determine whether the addition of DMSO introduces artefacts, the peptide length distribution and peptide motifs were examined. HLA class I and class II peptides displayed similar and expected peptide length distributions across both the control and DMSO groups, with lengths of 8–12 amino acids for HLA class I and 13–20 amino acids for HLA class II (Figure 2A,B). However, a detailed analysis of the HLA class I ligands revealed a distinct increase in shorter peptide sequences in the presence of DMSO (particularly 8mers and to a lesser extent 9mers) at the expense of longer peptides (Figure 2A). This phenomenon was even more pronounced in elastase-digested samples, wherein shorter ligands (7–9 amino acids) were observed to be proportionally more abundant than longer peptides (Figure 2C). In contrast, due to the overall longer peptide length preference, this effect was not observed in the HLA class II repertoire (Figure 2B), although analysis of the few potentially contaminant 7–11mer peptides in these samples revealed the same trend (Supplementary Figure S3). Taken together, these findings suggest that DMSO selectively improves the identification of shorter HLA peptide sequences.

### 3.3. DMSO Has Minimal Impact on Peptide Consensus Motifs

**Figure 3:**
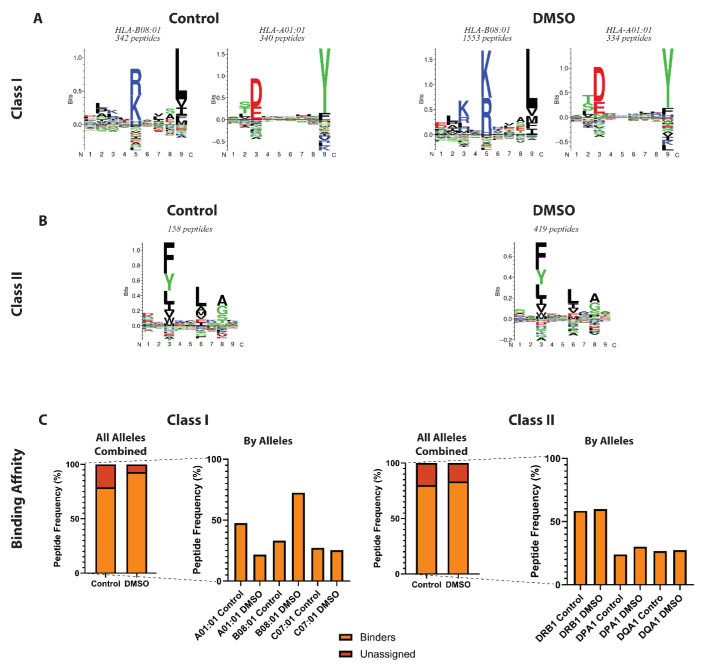
Binding motif and affinity of HLA class I and II peptides. (**A**,**B**) Gibbs clustering showing the motif for (**A**) class I (8–12mer) and (**B**) class II peptides (15mer), which were exclusively present in the control or DMSO group. Peptide numbers of each cluster are shown above the motifs. (**C**) Barchart summarising the percentage of predicted binder peptides for HLA class I and class II between in the absence and presence of DMSO, representing both the overall total (all alleles combined) on the left and allelic-level comparisons on the right.

To assess whether DMSO alters the peptide binding motifs or introduces sequence bias, unsupervised Gibbs clustering analyses were performed on unique peptides identified in both control and DMSO conditions. For the HLA class I peptides, two main clusters merged, corresponding to the canonical motifs of HLA-B*08:01 and HLA-A*01:01 alleles, which are representative of the B-LCL cell line used in this study (Figure 3A). Both groups combined showed characteristic enrichment of lysine (K) and arginine (R) at position 5 (P5), leucine (L) and tyrosine (Y) at position 9 (P9), and aspartic acid (D) and glutamic acid (E) at position 3 (P3). Interestingly, while the number of HLA-A01:01 peptides remained very similar between conditions, the number of peptides clustering to the HLA-B*08:01 motif increased approximately four-fold with DMSO (Figure 3A), which might explain the relatively higher proportion of arginine (R) and lysine (K) at P3 and P5 observed for HLA-B*08:01 with DMSO. Indeed, a deeper analysis of the peptide length within the HLA class I clusters revealed a pronounced 4–5-fold enrichment of 8- and 9mer peptides in the DMSO condition, consistent with the typical HLA-B*08:01 length distribution, whereas 8- and 9mers assigned to HLA-A*01:01 showed minimal changes relative to control (Supplementary Figure S4). HLA class II peptides displayed conserved motif features across both conditions, featuring phenylalanine (F) at position 3 and alanine (A) and glycine (G) at position 8, consistent with the DRB1*01:01 allele of the B-LCL cell line (Figure 3B). No major differences were observed in amino acid preferences at the N- or C-termini between control and DMSO-treated samples, indicating that DMSO does not introduce artefactual motif changes for both HLA class I and class II.

To further evaluate the impact of DMSO on peptide presentation, we applied the supervised binding prediction tools NetMHCpan-4.1 and NetMHCIIpan-4.1 [15,17]. In case of HLA class II, a slightly higher proportion of peptides were predicted to be genuine binders across all class II alleles in the presence of DMSO (Figure 3C). Interestingly, an allele-specific effect was observed for HLA class I. Notably, peptide identification associated with HLA-B*08:01 was enhanced in the presence of DMSO, whereas HLA-A01:01 and HLA-C07:01 showed modest reductions, consistent with clustering results (Figure 3A,C). A deeper analysis into this allele-specific effect revealed that HLA-B*08:01 predominantly presents 8mer peptides, whereas HLA-A*01:01 favours 9mers, consistent with previous reports [18] (Supplementary Figure S5). This observation aligns with our earlier findings that DMSO preferentially enhances the detection of shorter peptides (Figure 2C and Supplementary Figure S3), providing a plausible explanation for the preferential improvement in HLA-B*08:01 peptide detection and binding prediction.

Collectively, these results indicate that DMSO does not introduce major artefactual bias in sequence motif compositions and seems to increase the number of genuine peptide binders in an allele-specific manner. This is a critical attribute for immunopeptidomics and further supports the utility of DMSO in expanding and refining immunopeptidome profiling.

### 3.4. DMSO Enhances Ionisation Efficiency

**Figure 4:**
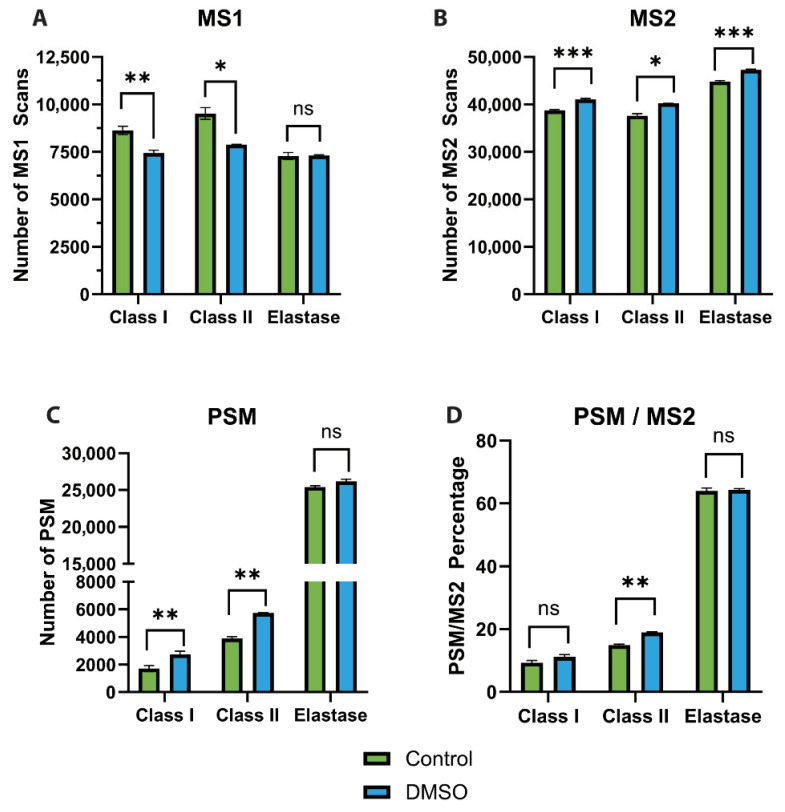
Number of precursor (MS1) and fragment ion (MS2) scans. (**A**,**B**) Bar charts showing the average number of (**A**) MS1 and (**B**) MS2 scans acquired across the three sample types—HLA class I, HLA class II, and elastase-digested peptides—under control (green) and DMSO (blue) conditions. (**C**,**D**) Bar charts showing the (**C**) number of peptide spectral matches (PSM) and (**D**) percentage ratio of PSM to number of MS2 scans—under control (green) and DMSO (blue) conditions. Statistical significance was assessed using Student’s *t*-test. ns: not significant; *: *p* < 0.05; **: *p* < 0.001; ***: *p* < 0.0001. Data represent the mean ± standard error of the mean (SEM) from three technical replicates (N = 3).

**Figure 5:**
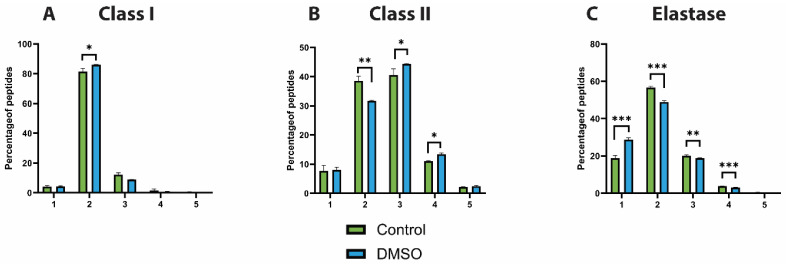
Charge distribution. Bar charts showing the charge distribution of the (**A**) HLA class I, (**B**) HLA class II and (**C**) elastase-digested peptides under control (green) and DMSO (blue) conditions. Statistical analysis was performed with the Student *t*-test. NS: non-significant, *: <0.05, **: <0.001 and ***: <0.0001. Supplementary Figures S8 and S9 provide detailed breakdowns by length. Data represent the mean ± standard error of the mean (SEM) from three technical replicates (N = 3).

To assess the impact of DMSO on mass spectrometric performance, precursor (MS1) and fragment ion (MS2) scan profiles were evaluated. In the presence of DMSO, a consistent and significant reduction in MS1 scans was observed for both HLA class I and II peptide samples, with minimal changes noted in the elastase-digested samples (Figure 4A). Interestingly, individual peptide signal intensities were generally higher with DMSO (Supplementary Figure S6), suggesting improved and possibly more stable ionisation efficiency. In contrast, the number of MS2 scans increased significantly across all sample types in the presence of DMSO (Figure 4B), reflecting the expected shift in instrument utilisation from MS1 to MS2 acquisition. This increase was accompanied by a higher number of peptide spectrum matches (PSMs), with increase of ~1.5 folds for HLA classes I and II and ~1.1 folds for elastase digests (Figure 4C), demonstrating that the additional MS2 scans translated into more peptide identifications. Furthermore, to verify that the reduction in MS1 scans was not caused by prolonged ion accumulation, we analysed mean injection times. Both MS1 and MS2 injection times showed slight decreases—with a more pronounced reduction at the MS1 level—in the presence of DMSO (Supplementary Figure S7). These findings indicate improved acquisition efficiency rather than slower cycle time. To further explore the basis of these improvements, charge state distributions were analysed, as they can directly influence MS2 acquisition and subsequent spectral quality [14]. DMSO induced a modest but consistent shift in charge states that correlated with peptide length. Shorter HLA class I peptides showed a slight increase in +2 charge states, while HLA class II peptides exhibited a rise in +3 and +4 charge states at the expense of doubly charged species, consistent with their longer lengths (Figure 5A,B). This trend was even more pronounced when examining peptides exclusively identified in either condition, where DMSO produced a larger increase across all charge states compared with control (Supplementary Figure S8). Notably, DMSO also enabled the detection of a distinct subset of +1-charged peptides that was entirely absent in the control condition, indicating improved detectability of low-charge precursors. Elastase-digested peptides, which are enriched in shorter sequences, displayed a marked increase in singly charged species (Figure 5C). Further stratification by peptide length confirmed that shorter peptides were more likely to acquire +1 or +2 charges, whereas longer peptides favoured +3 or +4 charges (Supplementary Figure S9), reflecting the expected charge–length relationship. Notably, elastase peptides displayed a charge distribution pattern intermediate to HLA classes I and II, consistent with their mixed length profile.

Beyond charge state and peptide length, we examined whether DMSO influenced other physicochemical properties, focusing on hydrophobicity given the use of a C18 reverse-phase column the potential influence of DMSO on other physicochemical properties was also examined. Notably, we focused on hydrophobicity, as our LC-MS/MS workflow uses reverse-phase chromatography, which separates peptides based on their hydrophobicity. Analysis of peptide hydrophobicity using the GRAVY score revealed a 15–25% increase in the proportion of hydrophilic peptides for both HLA classes I and II under DMSO treatment (Supplementary Figure S10). In contrast, elastase-digested peptides exhibited a slight increase in hydrophobic peptides, and this trend persisted even after filtering for shorter HLA-like peptides.

Collectively, these findings suggest that DMSO enhances ionisation efficiency of HLA-bound peptides by promoting favourable charge state distributions resulting in higher MS1 peptide intensities, which in turn results in more MS2 scans and an increase in PSMs.

### 3.5. DMSO Produces Higher-Quality MS2 Scans

**Figure 6:**
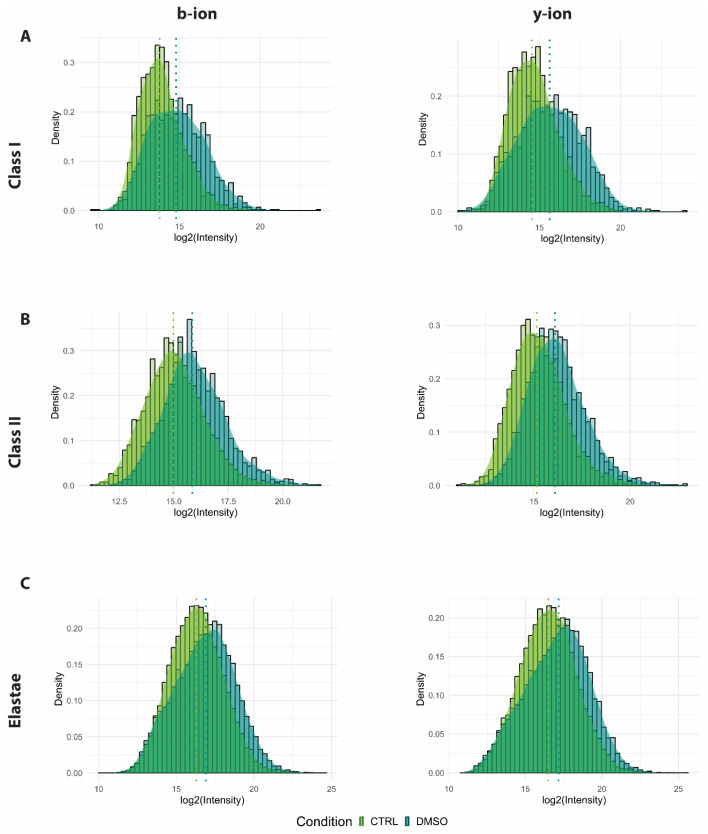
Fragment ion intensities (MS2) post addition of DMSO. The histogram shows the distribution of the b- and y-ion intensities in (**A**) HLA class I, (**B**) HLA class II, and (**C**) elastase-digested samples under control (green) and DMSO (blue) conditions. The log2 fold-change in the intensity values is shown on the x-axis, with the frequency displayed on the y-axis. The median values of the density curves are highlighted with dotted lines.

**Figure 7:**
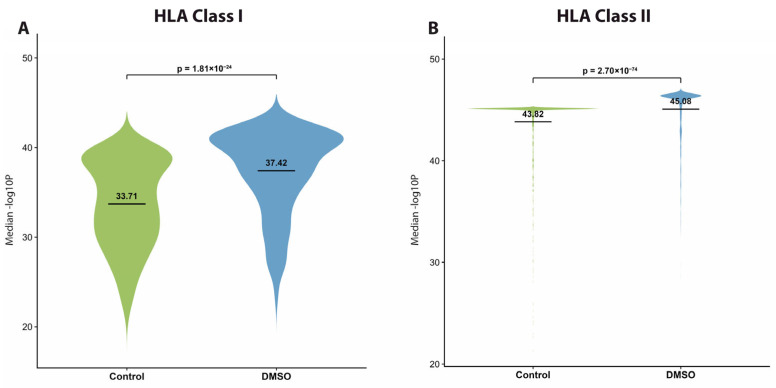
Peptide confidence score post addition of DMSO. Violin plot showing the median peptide identification confidence score (−log10P) for (**A**) HLA class I and (**B**) HLA class II under control (green) and DMSO (blue) conditions. Statistical analysis was performed with the Student *t*-test.

Building on the observed effects of DMSO on charge states and precursor ion intensities (Figure 5 and Supplementary Figures S6 and S9), we examined whether these changes translated into improved fragmentation (MS2) scan quality, particularly regarding the intensities of the b- and y-fragment ions. To evaluate this, we first analysed the PSM-to-MS2 scan ratio and observed a small, modest increase across all sample types, reaching statistical significance only for HLA class II peptides (Figure 4D). This trend suggests that MS2 scans acquired under DMSO conditions were generally of higher quality and more likely to yield successful peptide identifications. We then analysed the spectral intensities of b- and y-fragment ions as a proxy for MS2 spectral quality. The comparison revealed an average increase of at least 2 folds in the intensity of both b- and y-ions for HLA class I and class II peptides in the presence of DMSO (Figure 6A,B). This significant enhancement in ion intensities indicates a marked improvement in the quality of MS2 spectra, consistent with the previously observed increase in PSMs and MS2 scan counts (Figure 4B,C). As expected, elastase-derived peptides demonstrated an increase in ion intensities akin to that observed for HLA class I and class II peptides (Figure 6C). Moreover, to further support this observation, we analysed the peptide identification confidence score (−log10P) and observed a significant increase in peptide confidence score upon addition of DMSO for both HLA class I and class II (Figure 7). In summary, these findings strongly suggest that DMSO improves MS2 spectral quality, which likely contributes to the observed increase in PSMs.

## 4. Discussion

This study demonstrates that incorporating 5% DMSO into LC-MS/MS mobile-phase buffers can significantly improve peptide identification in immunopeptidomic workflows, with an observed increase of approximately 1.5 folds for a number of HLA peptides. This improvement can be attributed to multiple analytical dimensions, including ionisation efficiency, charge state distribution, and spectral quality, without introducing major sequence bias or compromising data integrity. Such enhancements are particularly relevant given the inherent challenges of immunopeptidomics, where peptides often lack basic residues and exhibit diverse physicochemical properties, making them difficult to ionise and fragment effectively.

A key outcome of this study was the consistent and reproducible increase in unique peptide identifications across HLA class I and class II. This reproducibility across replicates underscores the robustness of DMSO as an additive and suggests that its effect is systematic rather than stochastic. Importantly, while DMSO expanded the detectable immunopeptidome, the core repertoire remained intact, which is critical for comparative and longitudinal studies where biological consistency is essential. Furthermore, DMSO increased overall peptide coverage by approximately 40% for both HLA classes, highlighting its potential value for clinical and low-input samples. However, a subset of peptides was only detected under control conditions, indicating that some sequences may be missed when DMSO is added. This highlights the need for further optimisation of workflow parameters to maximise coverage, or alternatively, dual acquisition strategies, with and without DMSO, can be adopted to ensure comprehensive peptide coverage.

One of the most striking observations was the detection of shorter peptides suggesting that DMSO preferentially benefits peptides with lower charge states, which are typically underrepresented in standard immmunopeptidomics workflows. Charge state analysis confirmed a shift toward higher charge states, with shorter peptides acquiring +1 or +2 charges and longer peptides favouring an increase to +3 or +4. Importantly, Supplementary Figure S8 highlights a larger subset of +1-charged peptides detectable only in the presence of DMSO, demonstrating that DMSO improves the ionisation efficiency of precursors that would otherwise fall below the detection threshold. This increase in charge states likely contributes to improved ionisation efficiency, resulting in stronger MS1 signal intensities and better precursor selection for fragmentation. This observation aligns with established ionisation principles and highlights how DMSO’s supercharging effect is modulated by peptide length and physicochemical properties. Moreover, by improving the ionisation of shorter peptides, DMSO addresses a key limitation in immunopeptidomics, where such peptides often escape detection due to poor ionisation efficiency.

Additionally, an allele-specific analysis revealed that HLA-B*08:01 peptides, which are typically shorter, showed greater enhancement compared to HLA-A*01:01. This suggests that DMSO’s benefits may vary depending on allele-specific peptide length preferences, opening opportunities to fine-tune detection for specific alleles or peptide subpopulations. Motif analysis and binding predictions confirmed that DMSO does not introduce artefactual sequence bias, maintaining motif integrity while improving coverage. This is a critical observation for immunopeptidomics, where accurate motif representation underpins downstream applications such as epitope prediction and vaccine design.

Beyond increased in peptide identification, DMSO improved fragmentation quality, as evidenced by the approximately two-fold increase in b- and y-ion intensities. This enhancement in MS2 spectral quality facilitates more confident peptide assignments, particularly for low-abundance peptides that are often challenging to validate. Overall, these more intense fragment ions directly translate into higher-quality spectra and more confident PSMs, improving identification reliability and reinforcing the value of DMSO for identification accuracy. Taken together, these findings establish DMSO as a robust and broadly applicable additive for immunopeptidomics, particularly valuable for low-input or clinically derived samples where analytical sensitivity is critical. By improving electrospray ionisation, precursor charge state distribution, and MS2 spectral quality, DMSO addresses several longstanding technical limitations of immunopeptidomic workflows and enhances both the depth and reliability of peptide identification. Future studies should systematically assess the impact of DMSO on low-abundance ligands across diverse HLA alleles, including peptides presented by non-classical HLA molecules such as HLA-E and HLA-G, as well as samples derived from needle biopsies, rare proteoforms, and non-canonical open reading frames. In addition, integration of DMSO-enhanced workflows with DIA and PRM strategies may enable more comprehensive and high-throughput immunopeptidome profiling, with particular relevance for emerging ultra-low-input applications, including extremely rare cell populations and future single-cell immunopeptidomics, further accelerating translational applications in immunotherapy and biomarker discovery.

## Data Availability

The mass spectrometry proteomics data have been deposited to the ProteomeXchange Consortium via the PRIDE partner repository with the dataset identifier PXD072997.

## Supplementary Materials

The following supporting information can be downloaded at https://www.mdpi.com/article/10.3390/proteomes14010013/s1. Table S1. Excel containing peptides identified following PEAKS search. Table S2. Excel containing the binding affinity results from NETMHCpan4.1 and NETMHCIIpan4.1. Figure S1. Overlap between replicates: Venn diagram showing the overlap of the (A) HLA class I and (B) HLA class II replicated between control and DMSO condition. Figure S2. Peptide abundance rank: Graphs showing the rank abundance plots of the identified peptides in (A) HLA Class I, (B) HLA Class II, and (C) elastase-digested samples, which highlights overall increased detection levels in the presence of DMSO. Figure S3. HLA class II extended peptide length range: Bar charts showing the distribution of peptide lengths for HLA class II peptides focusing on the shorter 7–11mer peptides under control (green) and DMSO-treated (blue) conditions. Data represent the mean ± standard error of the mean (SEM) from three technical replicates (N = 3). Figure S4. Gibbs clustering peptide length distribution: Bar chart showing the HLA class I peptide length distribution within each Gibbs clusters from Figure 3A. Figure S5. Peptide Length Distribution of HLA-B08:01 and HLA-A01:01: Bar charts showing the distribution of peptide lengths for (A) HLA-B*08:01 and (B) HLA-A*01:01 under control (green) and DMSO (blue) conditions. The plots highlight the relative enrichment of shorter peptides in the presence of DMSO, particularly within HLA-B*08:01 samples. Figure S6. Peptide intensity: Volcano plots of the individual signal intensities of (A) class I, (B) class II and (C) elastase-digested peptides showing DMSO on the right and control on the left. Significantly regulated (2-fold change and *p* value ≤ 0.05) peptides are coloured in pink. Figure S7. MS1 and MS2 injection time: Bar chart showing the mean MS1 and MS2 injection between control and DMSO for HLA class I, HLA class II and elastase. Figure S8. Peptide exclusive to DMSO stratify by charge: Bar plots illustrating the distribution of charge states for peptides detected uniquely in control (green) or uniquely in DMSO (blue) for (A) HLA class I and (B) HLA class II. Figure S9. Stratified analysis of charge states by peptide length: Bar charts illustrating the correlation between peptide length and charge state preference for (A) HLA class I, (B) HLA class II, and (C) elastase-digested samples under control (green) and DMSO (blue) conditions, stratified by peptide length. Data represent the mean ± standard error of the mean (SEM) from three technical replicates (N = 3). Figure S10. Hydrophobicity profiles of peptides identified with and without DMSO: Distribution of peptide hydrophobicity scores based on the GRAVY scale for (A) HLA class I and (B) HLA class II peptides, showing an increase in hydrophilic peptides under DMSO treatment. (C) Hydrophobicity distribution for elastase-digested peptides, showing a slight increase in hydrophobic peptides with DMSO.

## Abbreviations

The following abbreviations are used in this manuscript:

ACN	Acetonitrile
B-LCL	B-lymphoblastoid Cell Line
DDA	Data Dependent Acquisition
DMSO	Dimethyl Sulfoxide
ESI	Electrospray Ionisation
HLA	Human Leukocyte Antigen
LC-MS/MS	Liquid Chromatography Tandem Mass Spectrometry
MHC	Major Histocompatibility Complex
MS	Mass Spectrometry
PSM	Peptide Spectral Match
TFA	Trifluoroacetic Acid
TSM	Tumour Stem Media
SDC	Sodium Deoxycholate
SEM	Standard Error of the Mean
