# Supplementary figures and images for: Dimethyl Sulfoxide Enhances HLA Peptide Identification
Source: Proteomes. 2026 Mar 13;14(1):13. doi: 10.3390/proteomes14010013 (PMC13030240; doi:10.3390/proteomes14010013)

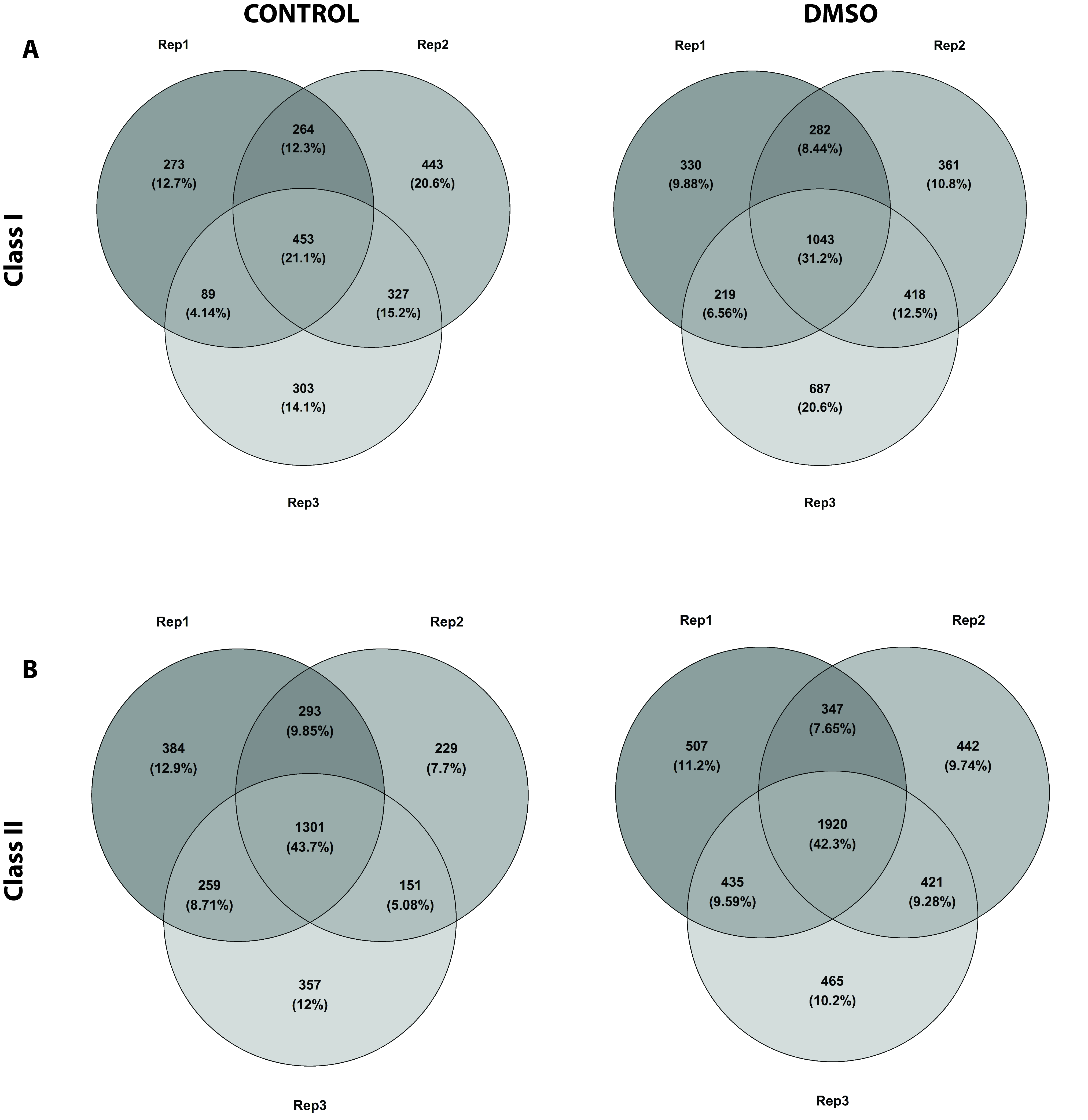

Supplement: Supplementary file 1 [file proteomes-14-00013-s001.zip › Supplementary Figure S1.tif]

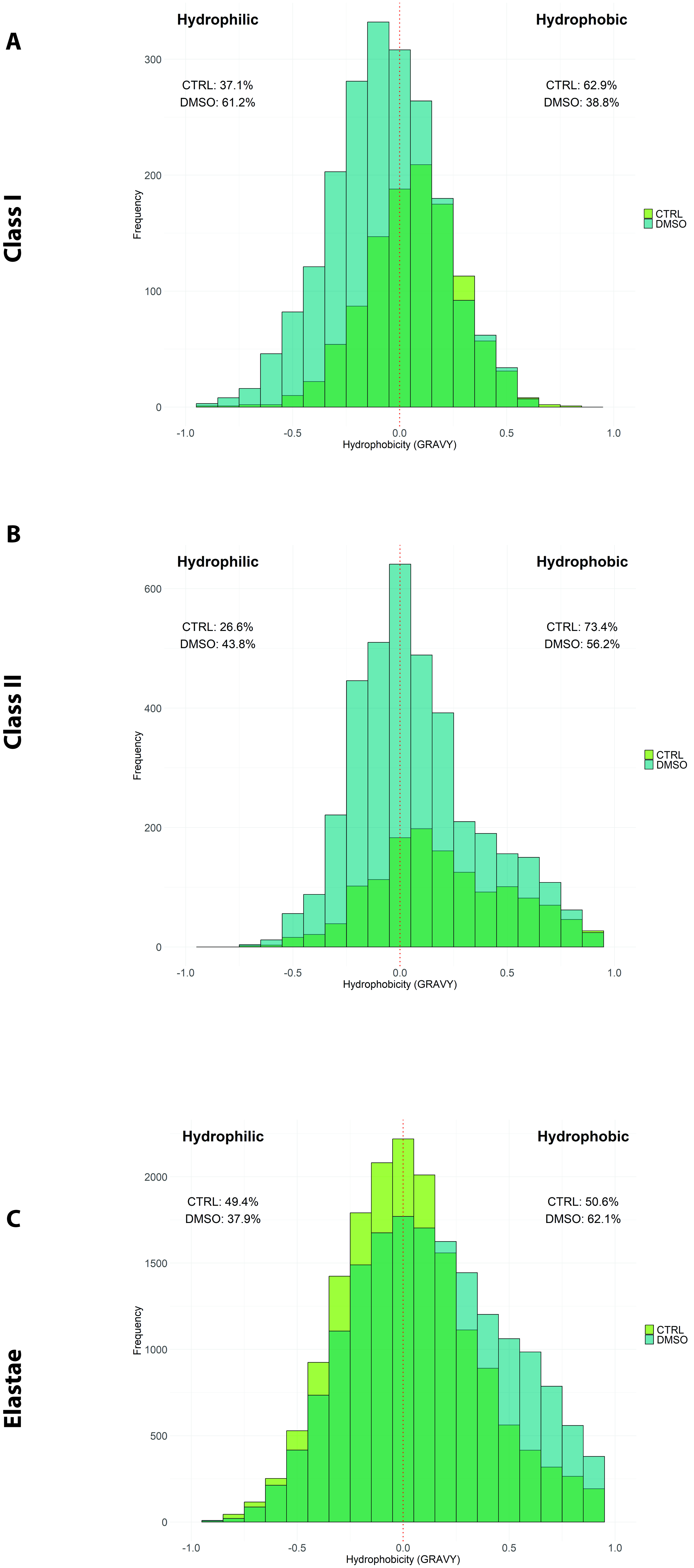

Supplement: Supplementary file 1 [file proteomes-14-00013-s001.zip › Supplementary Figure S10.tif]

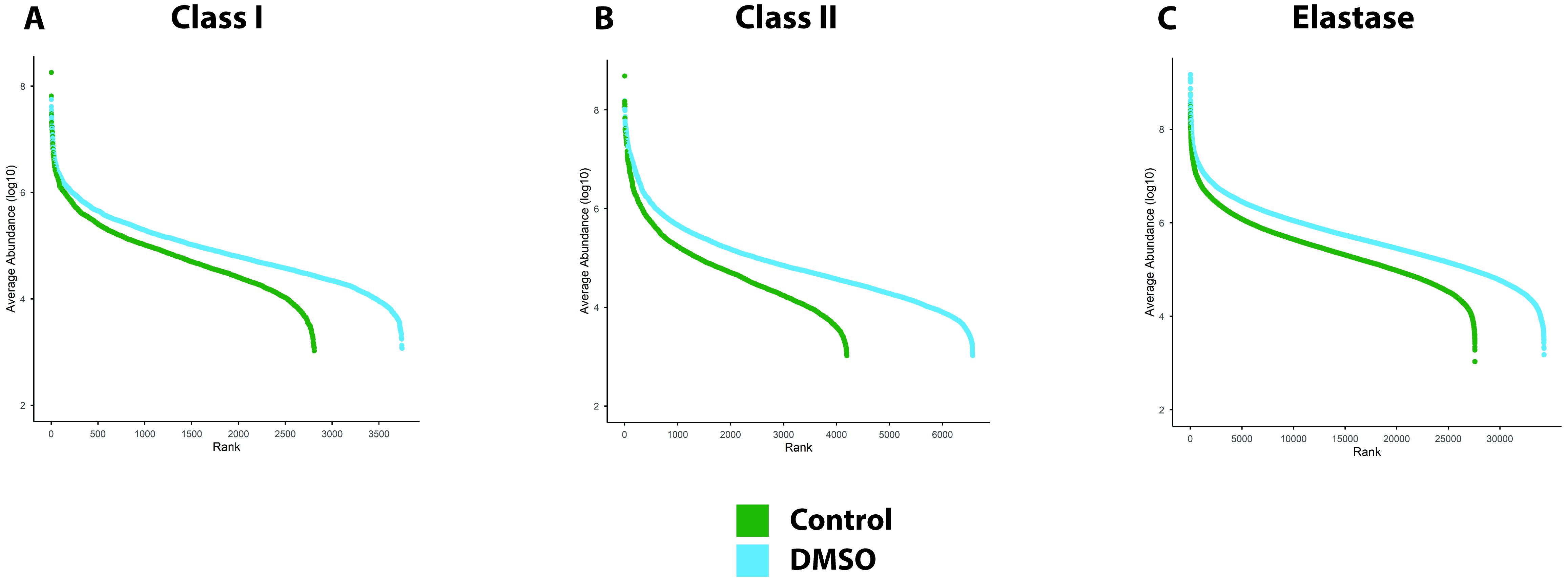

Supplement: Supplementary file 1 [file proteomes-14-00013-s001.zip › Supplementary Figure S2.tif]

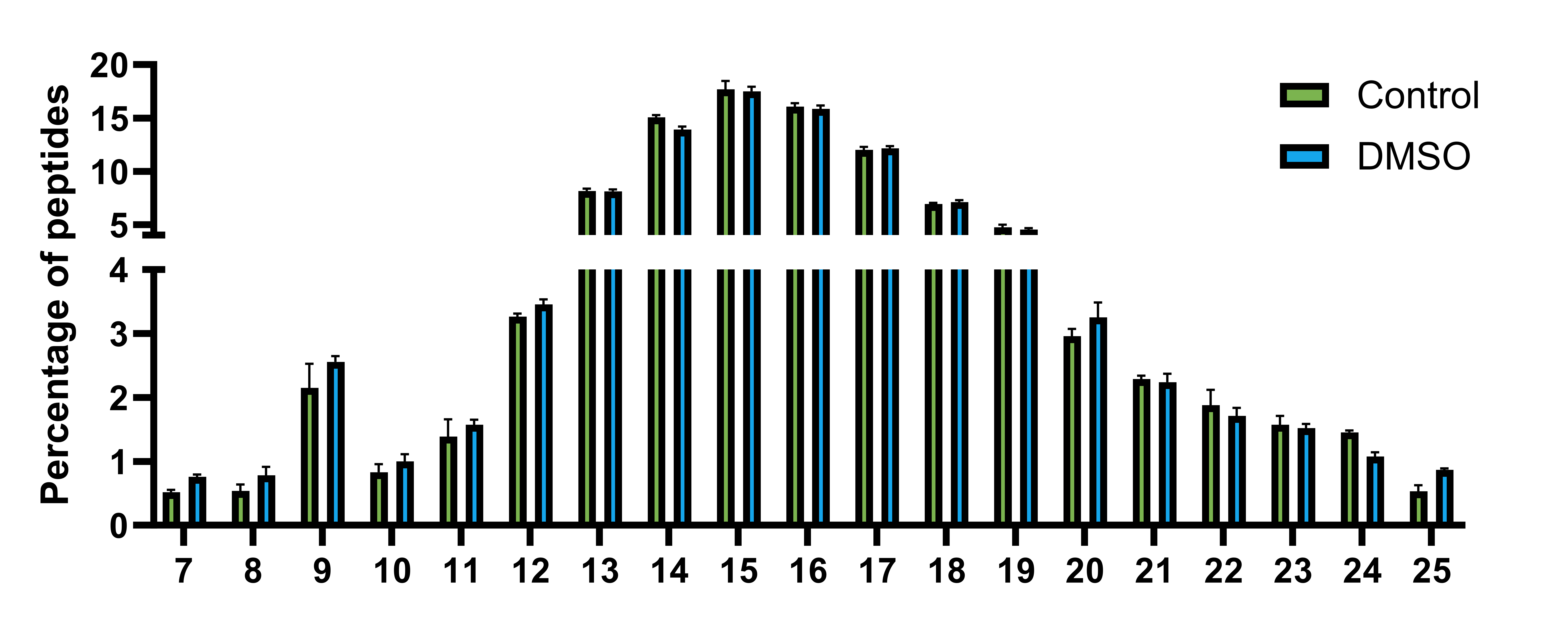

Supplement: Supplementary file 1 [file proteomes-14-00013-s001.zip › Supplementary Figure S3.tif]

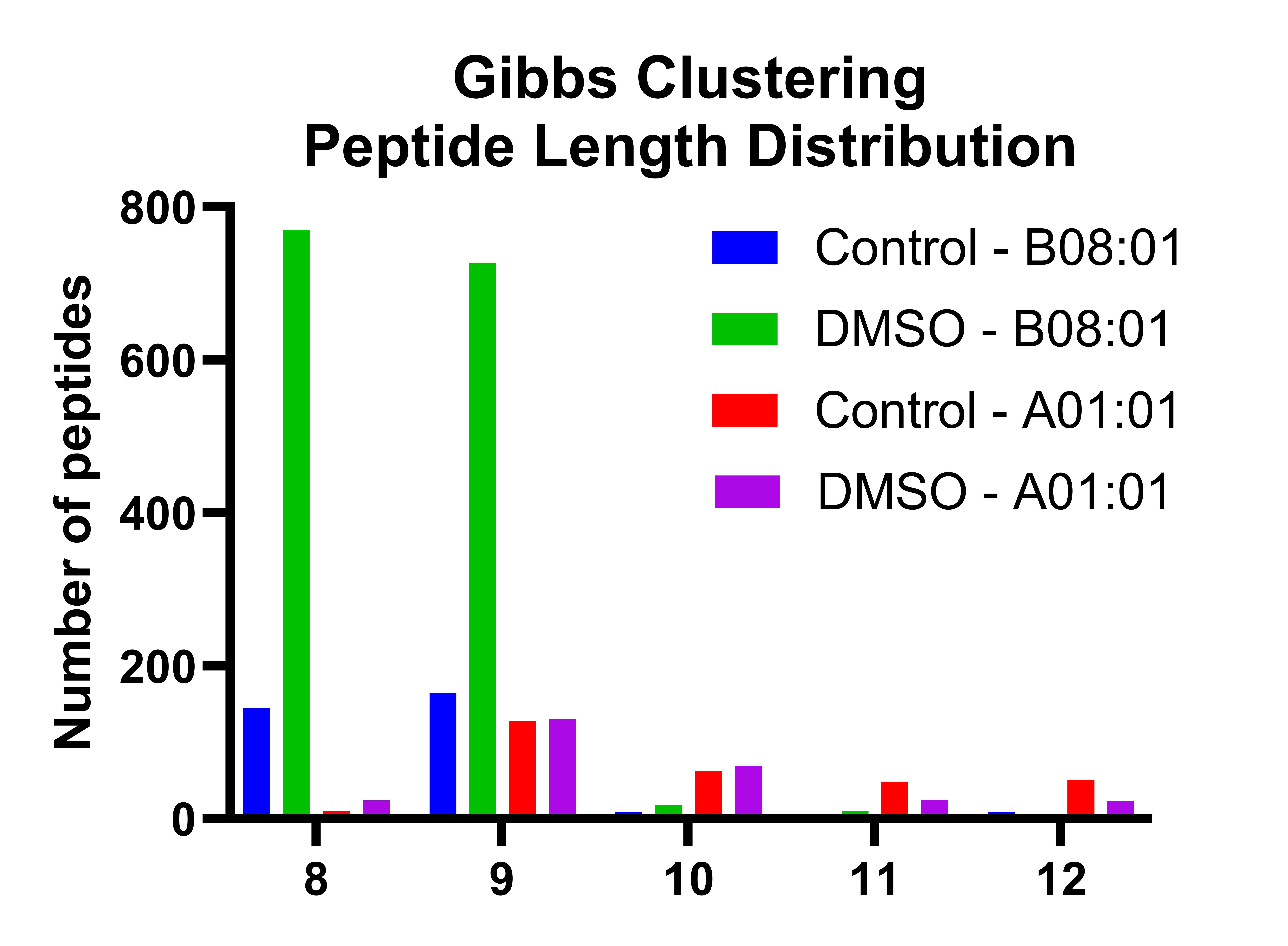

Supplement: Supplementary file 1 [file proteomes-14-00013-s001.zip › Supplementary Figure S4.tif]

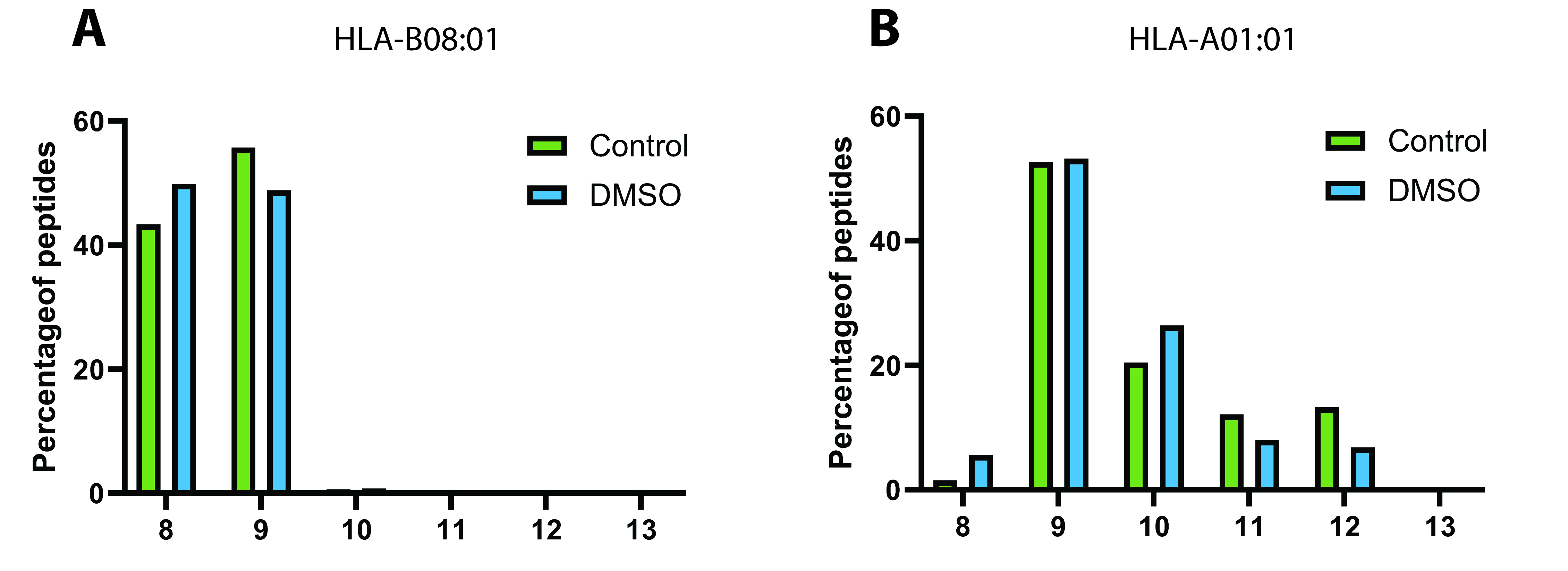

Supplement: Supplementary file 1 [file proteomes-14-00013-s001.zip › Supplementary Figure S5.tif]

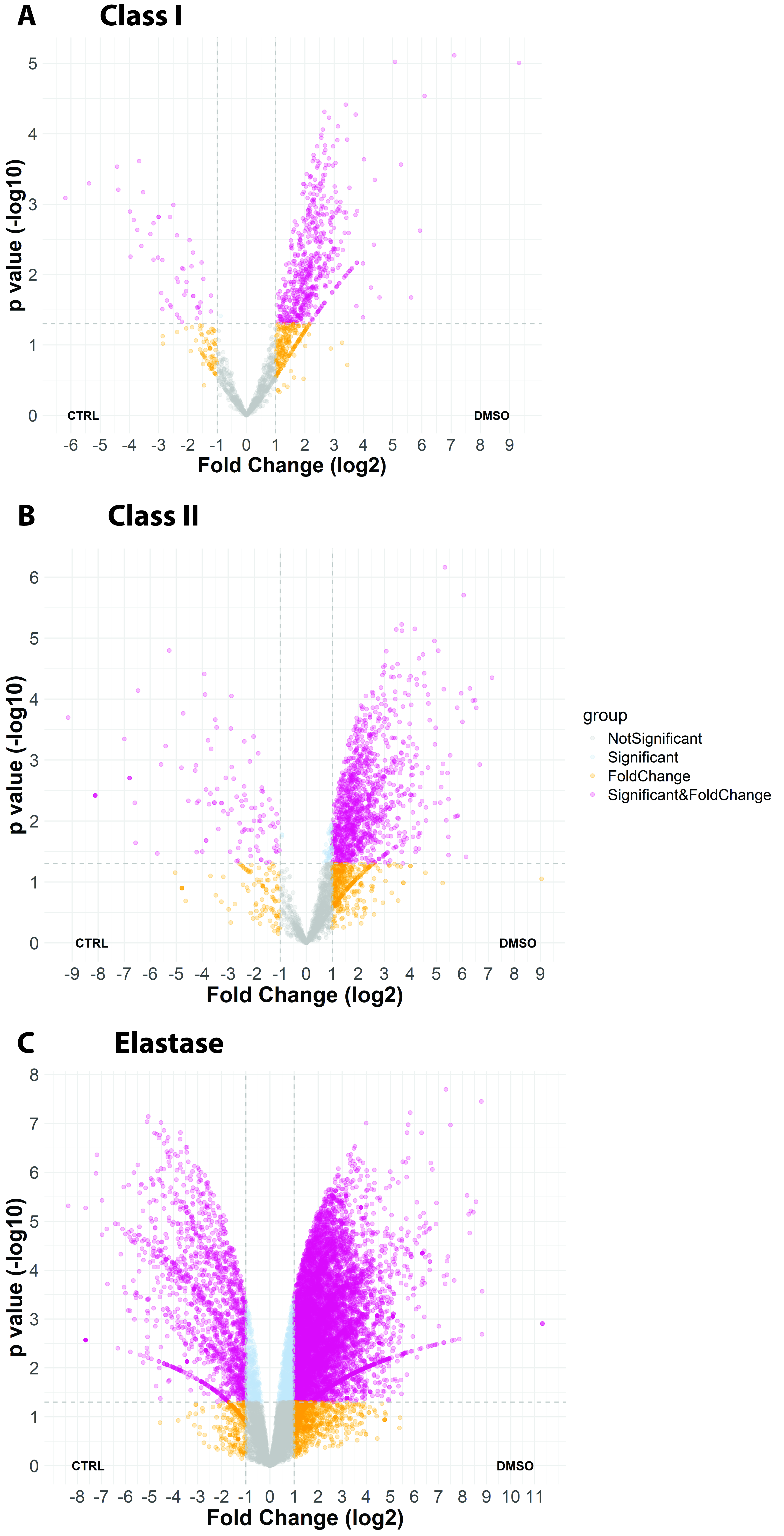

Supplement: Supplementary file 1 [file proteomes-14-00013-s001.zip › Supplementary Figure S6.tif]

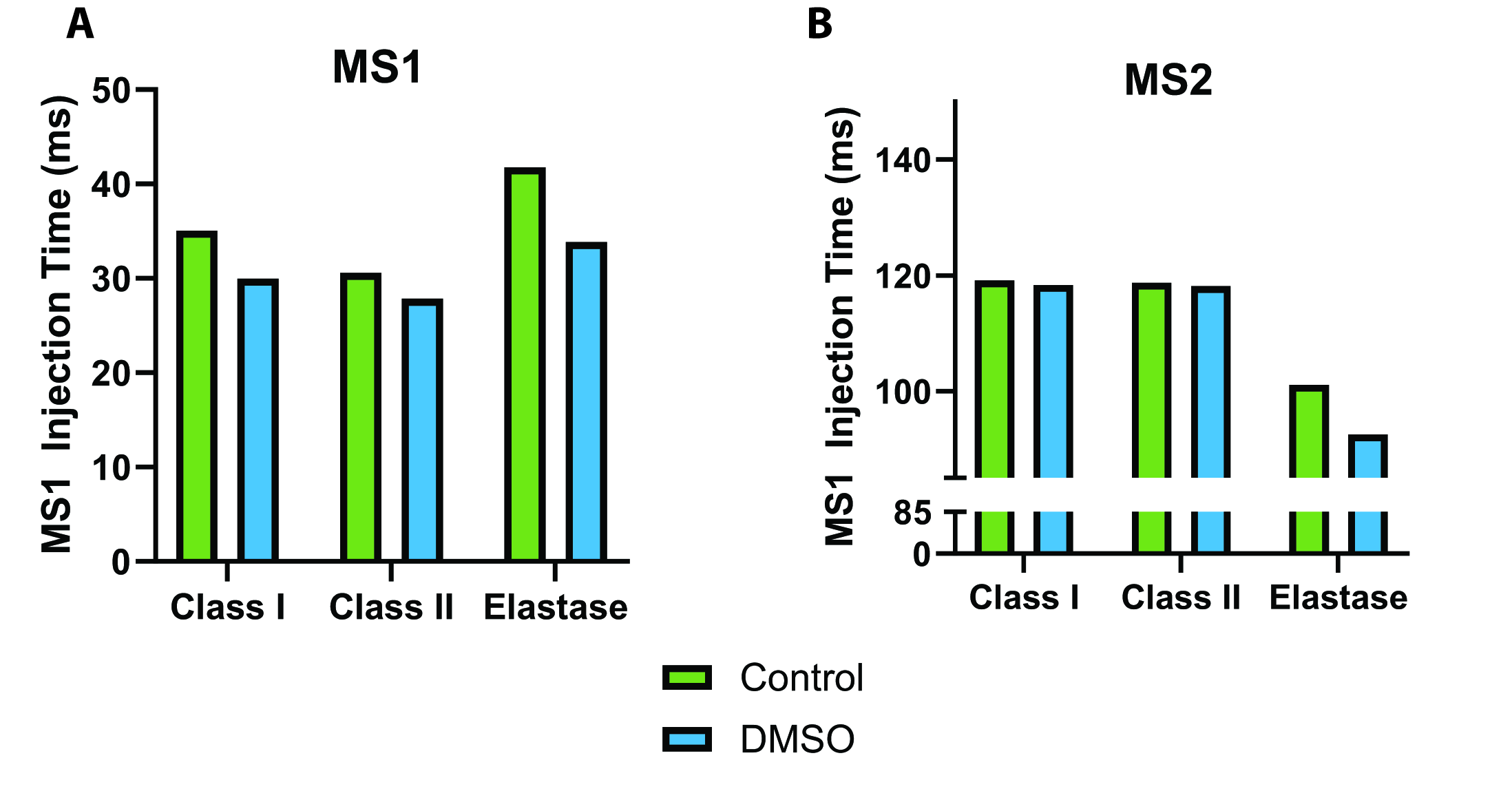

Supplement: Supplementary file 1 [file proteomes-14-00013-s001.zip › Supplementary Figure S7.tif]

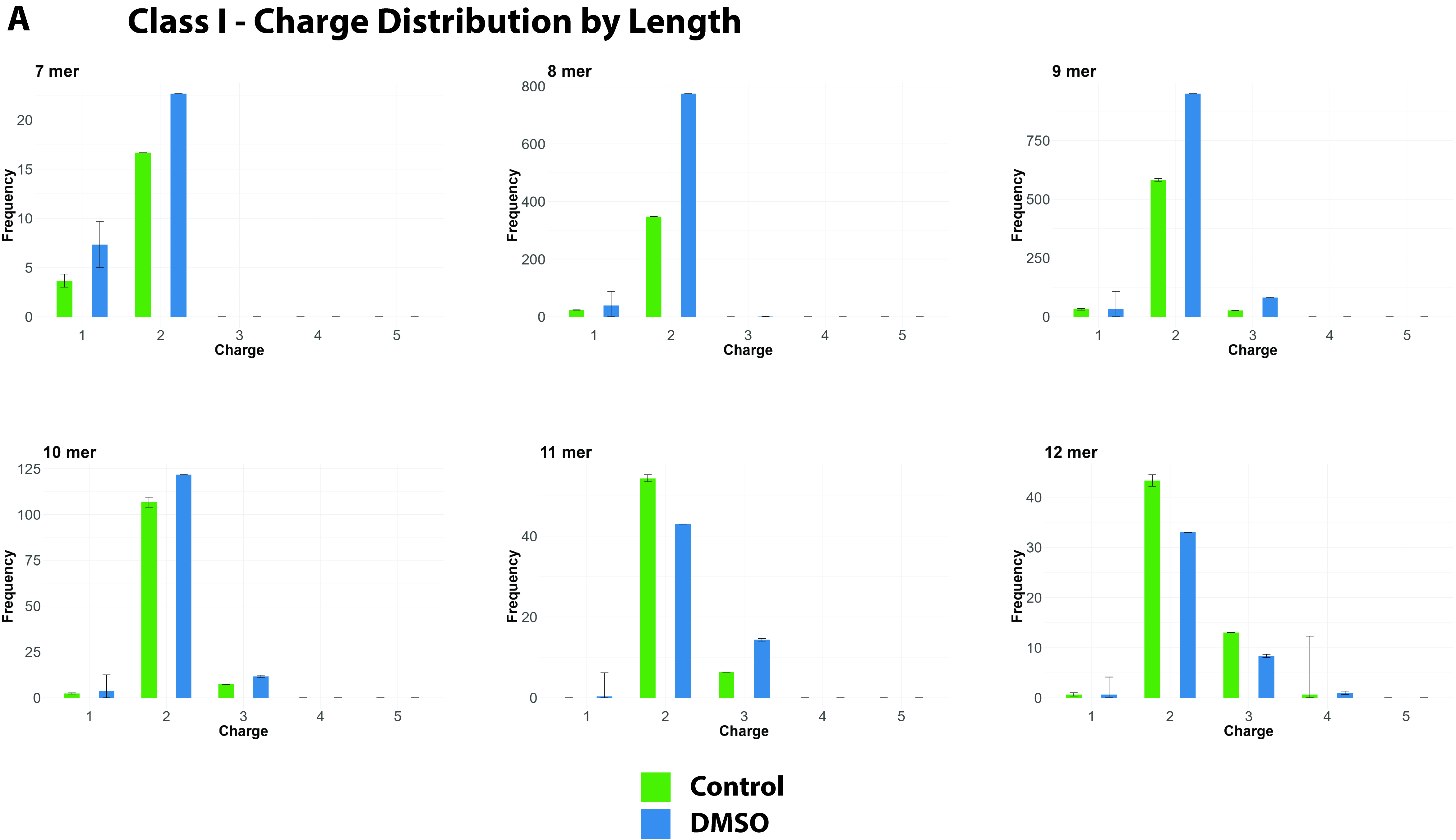

Supplement: Supplementary file 1 [file proteomes-14-00013-s001.zip › Supplementary Figure S9A.tif]
